# Postpartum-Specific Vital Sign Reference Ranges

**DOI:** 10.1097/AOG.0000000000004239

**Published:** 2021-01-05

**Authors:** Lauren J. Green, Rebecca Pullon, Lucy H. Mackillop, Stephen Gerry, Jacqueline Birks, Dario Salvi, Shaun Davidson, Lise Loerup, Lionel Tarassenko, Jude Mossop, Clare Edwards, Rupert Gauntlett, Kate Harding, Lucy C. Chappell, Marian Knight, Peter J. Watkinson

**Affiliations:** Nuffield Department of Clinical Neurosciences, the Institute of Biomedical Engineering, Department of Engineering Science, the Nuffield Department of Women's & Reproductive Health, and the Centre for Statistics in Medicine, University of Oxford, Oxford, United Kingdom; the Department of Anaesthesia, Wellington Hospital, Wellington, New Zealand; and Guy's and St Thomas' NHS Foundation Trust and the Department of Women and Children's Health, King's College, London, and the National Perinatal Epidemiology Unit and the Oxford National Institute for Health Research Biomedical Research Centre, Nuffield Department of Clinical Neurosciences, University of Oxford, Oxford, United Kingdom.

## Abstract

To estimate normal ranges for postpartum maternal vital signs.

**METHODS:**

We conducted a multicenter prospective longitudinal cohort study in the United Kingdom. We recruited women before 20 weeks of gestation without significant comorbidities and with accurately dated singleton pregnancies. Women recorded their own blood pressure, heart rate, oxygen saturation and temperature daily for 2 weeks postpartum. Trained midwives measured participants' vital signs including respiratory rate around postpartum days 1, 7, and 14.

**RESULTS:**

From August 2012 to September 2016, we screened 4,279 pregnant women; 1,054 met eligibility criteria and chose to take part. Postpartum vital sign data were available for 909 women (86.2%). Median, or 50th centile (3rd–97th centile), systolic and diastolic blood pressures increased from the day of birth: 116 mm Hg (88–147) and 74 mm Hg (59–93) to a maximum median of 121 mm Hg (102–143) and 79 mm Hg (63–94) on days 5 and 6 postpartum, respectively, an increase of 5 mm Hg (95% CI 3–7) and 5 mm Hg (95% CI 4–6), respectively. Median (3rd–97th centile) systolic and diastolic blood pressure returned to 116 mm Hg (98–137) and 75 mm Hg (61–91) by day 14 postpartum. The median (3rd–97th centile) heart rate was highest on the day of birth, 84 beats per minute (bpm) (59–110) decreasing to a minimum of 75 bpm (55–101) 14 days postpartum. Oxygen saturation, respiratory rate, and temperature did not change in the 2 weeks postbirth. Median (3rd–97th centile) day-of-birth oxygen saturation was 96% (93–98). Median (3rd–97th centile) day-of-birth respiratory rate was 15 breaths per minute (10–22). Median (3rd–97th centile) day-of-birth temperature was 36.7°C (35.6–37.6).

**CONCLUSION:**

We present widely relevant, postpartum, day-specific reference ranges which may facilitate early detection of abnormal blood pressure, heart rate, respiratory rate, oxygen saturation and temperature during the puerperium. Our findings could inform construction of an evidence-based modified obstetric early warning system to better identify unwell postpartum women.

**CLINICAL TRIAL REGISTRATION:**

ISRCTN, 10838017.

## Abstract 2

Postpartum-specific reference ranges for blood pressure, heart rate, respiratory rate, oxygen saturation, and temperature may facilitate early identification of unwell postpartum women.

Most maternal deaths occur postpartum, either within 24 hours of birth (17% in the United States^1^; 25% in the United Kingdom^2^; 50% worldwide^3^) or in the subsequent 6 weeks (40% in the United States,^1^ 45% in the United Kingdom^2^). The postpartum period often receives less attention than antenatal and intrapartum care.^3^ The leading direct causes of postpartum pregnancy-related mortality are all associated with abnormal vital signs (venous thromboembolism, sepsis, and postpartum hemorrhage in the United States and the United Kingdom^1,4^ and gestational hypertensive disorders worldwide).^5^ Identification of physiologic deterioration postpartum needs improvement,^4^ but normal vital sign ranges are poorly defined.^6^ Previous studies usually report values on one or two occasions often several weeks postpartum.^7–14^ Daily data are rare, often small-scale, and outdated (Walters BNJ, Walters T. Hypertension in the puerperium [letter]. Lancet 1987;2(8554):330. doi: 10.1016/s0140-6736(87)90912-3).^15^ Fewer data exist for other vital signs postpartum.^6^ Recommended vital signs ranges in national reports (United Kingdom,^16^ Ireland,^17^ United States^18^), and modified obstetric early warning scores thresholds^19–22^ are based on expert opinion, with wide national and international variation. Thresholds used during pregnancy are used after birth, without accommodating changes for postpartum maternal physiology. Outside pregnancy, the use of robust estimates of vital sign distributions to generate early warning scores is reliable.^23,24^ The approach may be particularly useful postpartum, where event rates are low. The primary objective of the 4P (Pregnancy Physiology Pattern Prediction) study was to develop a database of prospective vital sign measurements using standardized measurement techniques throughout pregnancy and the first 2 weeks postpartum.^25,26^ We included a pragmatic “low-risk” population, representative of women who would be monitored using a modified obstetric early warning score derived from the normal vital sign ranges obtained. We derived estimates of population distributions and associated centiles from this database.

## METHODS

This work is reported following the STROBE (Strengthening the Reporting of Observational Studies in Epidemiology) guidelines.^27^ We registered the study (https://doi.org/10.1186/ISRCTN10838017). Detailed methods are published in the protocol.^26^ We conducted a multicenter, longitudinal, observational, cohort study across three U.K. centers. We collected vital sign data during the antenatal,^25^ intrapartum, and postpartum periods. Here we present postpartum data.

Recruitment commenced August 2012 with vital sign collection completed August 2017. The study started in Oxford as a substudy of the INTERBIO-21st Fetal Study, approved by Oxford South Central C Research Ethics Committee (REC:08/H0606/139),^28,29^ and expanded to include two additional centers (Newcastle and London; with approval granted by South East Coast–Brighton and Sussex Research Ethics Committee REC:14/LO/1312) continuing after completion of INTERBIO-21st (December 2015).

We approached women before 20 weeks of gestation. Eligible women were aged 16 years or older, with a singleton pregnancy, and within category one of the American Society of Anesthesiologists’ classification of physical status before pregnancy (“a normal healthy patient without any clinically important comorbidity and without clinically significant past or present medical history”^30^). Gestational age was determined by ultrasound measurement of crown–rump length before 14 weeks of gestation. Full eligibility criteria are in Appendix 1, available online at http://links.lww.com/AOG/C171. Participants provided informed written consent and could withdraw from the study at any time.

The primary outcome for this substudy was postpartum-specific reference ranges comprising vital sign centile distributions for the first 2 weeks of the puerperium. We assessed differences in self- and clinician-taken vital sign measurements.

We previously found no clinically meaningful differences between first and second measures of vital signs in pregnancy,^25^ so did not collect duplicate readings during the postpartum study period. We previously showed the performance of the devices used to measure each vital sign remained unchanged throughout the study.^25^

Trained research midwives provided instruction in the use of home monitoring equipment to participants around birth, using study standard operating procedures.^26^ Participating women measured and recorded daily vital sign data for four physiologic parameters: blood pressure, heart rate, oxygen saturation, and temperature until day 14 after birth using standardized equipment. Blood pressure was measured with an automated blood pressure monitor validated for use in pregnancy (Microlife 3BT0-A (2)/WatchBP Home). Heart rate and oxygen saturation were measured with a Bluetooth-enabled pulse oximeter (WristOx2 3150). Temperature was measured with a tympanic thermometer (Genius 2). Additionally, trained research midwives collected vital sign data following study standard operating procedures at home visits on up to three occasions after birth^26^ for five physiologic parameters: blood pressure, heart rate, oxygen saturation, temperature, and respiratory rate. Trained midwives also collected day 0 observations when visiting mothers waiting to be discharged to provide home monitoring equipment. For both participant and midwife-taken vital signs, heart rate, oxygen saturation values, and the photoplethysmography waveform were automatically transmitted via Bluetooth to an Android tablet computer (Samsung Galaxy Tab 4.0), with blood pressure and temperature inputted manually. Midwives measured respiratory rate using two methods: by observing chest wall movement over a 15-second period and by tapping in time with observed respiratory rate for 1 minute using a software application on the Android tablet computer (October 2015 onward, to explore whether this method was more reliable than the 15-second method). We also undertook an exploratory analysis to ascertain whether respiratory rate could be extracted from the photoplethysmography waveform recorded by the pulse oximeter.

Participants knew that self-collected vital signs were not reviewed in real time. It remained the participant’s responsibility to seek assistance if they felt unwell. Vital sign measurements taken for the study were not included in the clinical record and were not communicated to the clinical team unless blood pressure reached predefined values (systolic higher than 140 mm Hg or diastolic higher than 90 mm Hg) according to study standard operating procedures (Appendix 2, available online at http://links.lww.com/AOG/C171). Research midwife coordinators performed frequent site visits to carry out midwife training and address any recruitment and equipment issues.

We collected demographic information (age, height, weight, self-reported ethnicity, number of previous pregnancies, smoking status), past medical and obstetric history, current health status, pregnancy-related health and current medications at the initial assessment. Ethnicity was defined by the participant at the baseline visit according to classification by the National Institute for Health and Care Excellence, as referenced in our protocol.^26^ The option not to give ethnic group information was available to every participant. Ethnicity was collected to allow the generalizability of our population to be considered. At each follow-up appointment, we collected smoking status, current health status, pregnancy-related health and current medications. We extracted medical and obstetric history from the participants’ notes.

We published our sample size determination^26^ (and Appendix 3, available online at http://links.lww.com/AOG/C171). In brief, a sample size of 1,000 women would achieve an SE of 0.05*SD at the 2.5th and 97.5th centiles, and even greater precision at the less extreme centiles. Adequate precision was also met for any subgroup analysis; for example, a sample size of 300 women would achieve an SE of 0.1*SD at the 2.5th and 97.5th centiles.

We included vital sign data from all participants in the primary analysis (including participants who became lost to follow-up or had missing measures). Once enrolled, we did not exclude women who developed conditions that might affect their vital signs (to generate a pragmatic, representative sample of postpartum women and maximize the clinical applicability of centiles generated). We constructed smoothed centiles for systolic and diastolic blood pressure, heart rate, oxygen saturation, and temperature by day postpartum. We constructed postpartum-specific reference ranges comprising smoothed centiles for vital sign distribution (3rd, 10th, 50th, 90th, and 97th centiles as used by the World Health Organization Multicentre Growth Reference Study,^31,32^ with corresponding 95% CIs) for all women. We followed the statistical methods used in the INTERGROWTH-21st Project for fetal growth^33–36^ to generate postpartum day–specific centiles. We explored different statistical methods to achieve the best fit to the data (see Appendix 3, http://links.lww.com/AOG/C171). As respiratory rate was only recorded at midwife visits (meaning sufficient data for smoothed centiles would not be expected), we constructed empirical centiles based on grouping the data into four periods of 3.5 days, with results plotted at the mid-point of each period.

Where both self- and clinician-taken vital sign measurements were recorded within an hour, we assessed agreement using the Bland-Altman method. We pooled data when there was no substantial bias and limits of agreement were adequate. Where two sets of vital sign measurements were recorded by the same method (usually two sets of self-taken measurements) within an hour, we used the mean of the two values in our analyses.

We conducted a predefined subgroup analysis of the effect of parity on the postpartum reference ranges. To explore whether limiting the population to those of optimal health would affect results, we defined a “restrictive” population of women aged younger than 40 years with body mass indexes (BMIs, calculated as weight in kilograms divided by height in meters squared) between 18.5 and 29.9 who did not smoke and did not have comorbidities. In this “restrictive” population, we excluded measures from women who developed a severe maternal condition during pregnancy (severe preeclampsia; hemolysis, elevated liver enzymes, and low platelet count [HELLP] syndrome; or eclampsia, as defined in the study protocol^26^), in line with previous work.^37^ We conducted a post hoc analysis using these definitions to compare with the analysis of the full “pragmatic” population. We also undertook post hoc analyses of the effect of epidural and epidural or anesthesia on postpartum reference ranges.

## RESULTS

**Fig. 1.**
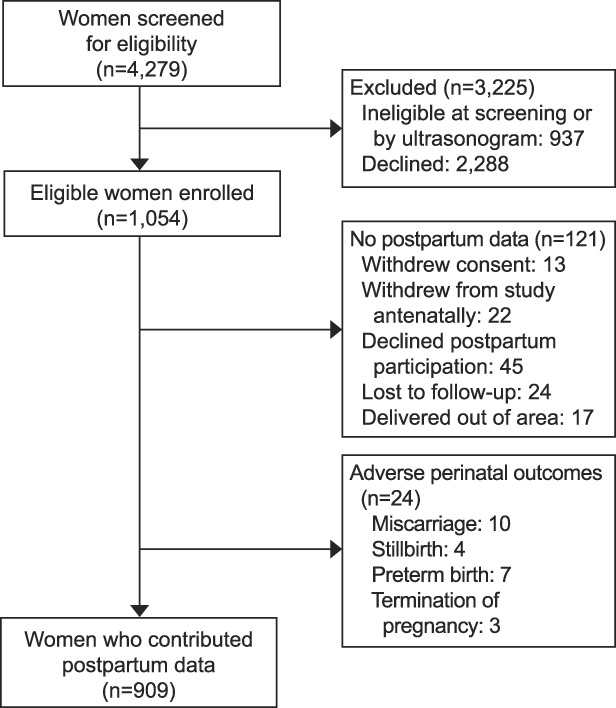
Flowchart of participants in the study. Green. Postpartum Vital Sign Reference Ranges. Obstet Gynecol 2021.

We screened 4,279 women between August 1, 2012, and December 28, 2016, of whom 1,054 agreed to take part. A cohort of 909 women contributed postpartum vital sign data (Fig. 1). Study cohort demographic characteristics were similar across sites (Table 1). At the first antenatal visit mean (SD) gestational age was 13.2 (2.5) weeks; maternal age 32.2 (4.7) years; BMI 24.8 (4.7) and 44.9% (408/909) were nulliparous. Maternal characteristics were similar in the postpartum cohort to those of all women enrolled (Appendix 4, available online at http://links.lww.com/AOG/C171).^25^

**Table 1.**
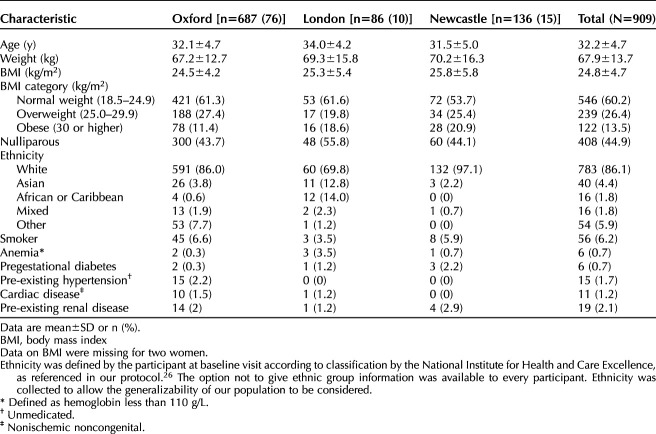
Baseline Maternal Characteristics

The median number of times per woman where vital signs were recorded was 11 (interquartile range 8 to 13). In total, 9,621 sets of vital sign data were recorded. Of these, blood pressure was recorded nearly always (9,534/9,621, 99%), heart rate 96% (9,221/9,621), oxygen saturation 96% (9,224/9,621) and temperature 97% (9,347/9,621). Respiratory rate was recorded at 92% of midwife visits (1,535/1,600 visits up to day 14). An abnormal blood pressure recording necessitating referral to the woman’s usual clinical team occurred at 0.03% of visits (3/9,621 observations; 3/909 women). Table 2 details pregnancy outcomes and perinatal events within the study cohort.

**Table 2.**
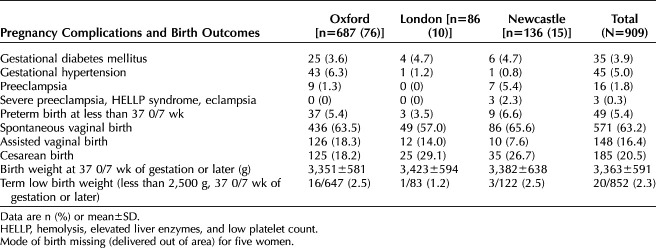
Pregnancy Complications and Perinatal Outcomes

**Fig. 2.**
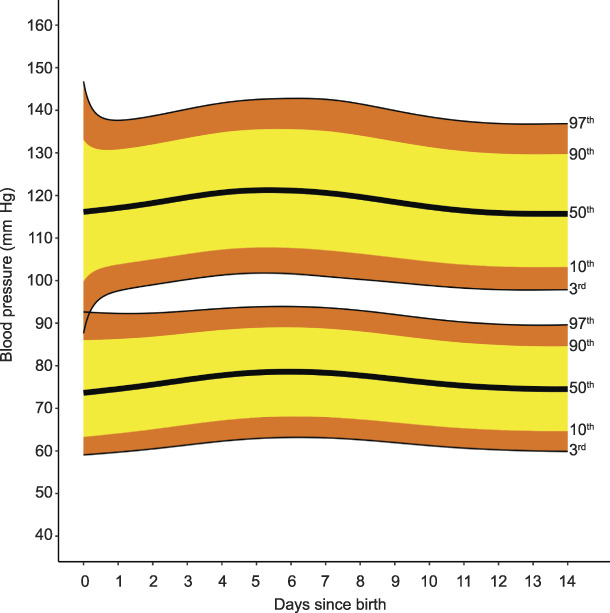
Smoothed centiles for systolic blood pressure (*upper line and centiles*) and diastolic blood pressure (*lower line and centiles*) in mm Hg. Day 0 indicates day of birth. Green. Postpartum Vital Sign Reference Ranges. Obstet Gynecol 2021.

**Fig. 3.**
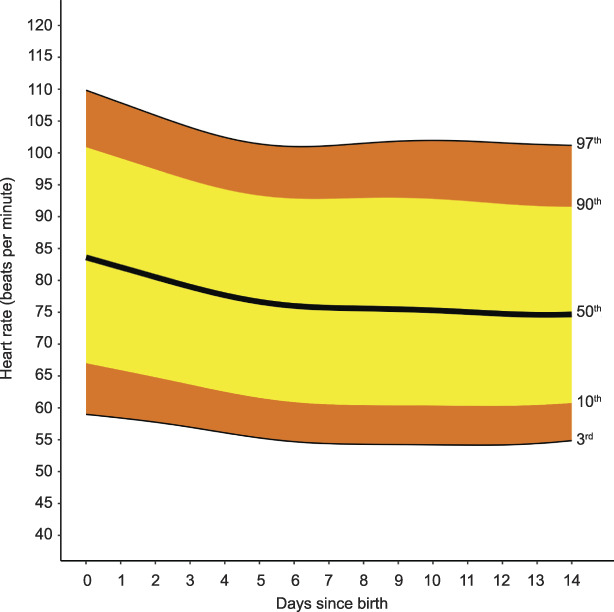
Smoothed centiles for heart rate in beats per minute. Day 0 indicates day of birth. Green. Postpartum Vital Sign Reference Ranges. Obstet Gynecol 2021.

The Bland-Altman plots did not show significant bias between self- and clinician-taken vital sign recordings (Appendix 5, available online at http://links.lww.com/AOG/C171). Therefore, we pooled data from the two groups for systolic and diastolic blood pressure, heart rate, oxygen saturation and temperature. Systolic blood pressure increased from the day of birth (day 0), from a median, or 50th centile, of 116 mm Hg (3rd–97th centile 88–147) to a maximum median of 121 mm Hg (3rd–97th centile 102–143) 5.4 days postpartum, an increase of 5 mm Hg (95% CI 3–7). Systolic blood pressure then decreased to a nadir of median 116 mm Hg (3rd–97th centile 98–137) 14 days postpartum, a difference of −6 mm Hg (95% CI −6 to −5) from maximum to minimum systolic blood pressure. Diastolic blood pressure was lowest on the day of birth: median, or 50th centile, 74 mm Hg (3rd to 97th centile 59–93). Diastolic blood pressure rose to a maximum median of 79 mm Hg (3rd–97th centile 63–94) 6 days postpartum, a difference of 5 mm Hg (95% CI 4–6) from minimum to maximum diastolic blood pressure. Diastolic blood pressure subsequently decreased: median 75 mm Hg (3rd–97th centile 60–90) 14 days postpartum, a change of −4 mm Hg (95% CI −5 to −3) (Fig. 2). The median, or 50th centile, heart rate was highest on the day of birth: 84 beats per minute (bpm) (3rd–97th centile 59–110). Median heart rate decreased progressively to the 7th day after birth: day 7 median 76 bpm (3rd–97th centile 54–101) (Fig. 3), a difference of −8 bpm (95% CI −11 to −5). There was no further significant change in heart rate by day 14, with a median value of 75 bpm (3rd–97th centile 55–101), a difference of −0.5 bpm (95% CI −1.9 to 0.8).

**Fig. 4.**
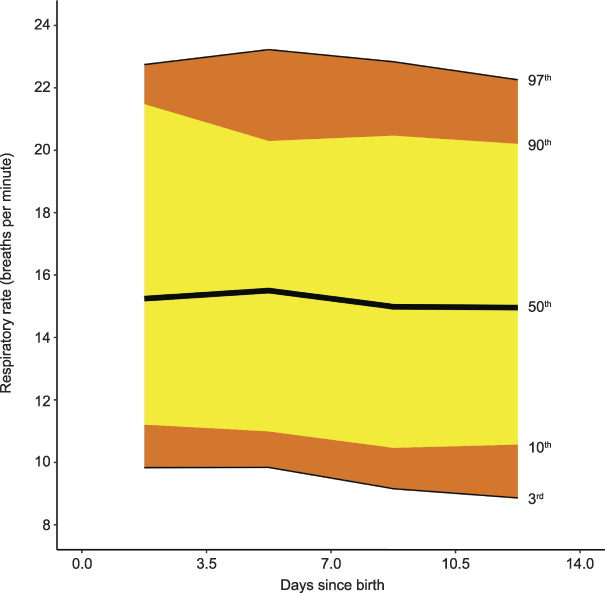
Smoothed centiles for respiratory rate (breaths per minute). Day 0 indicates day of birth. Green. Postpartum Vital Sign Reference Ranges. Obstet Gynecol 2021.

**Fig. 5.**
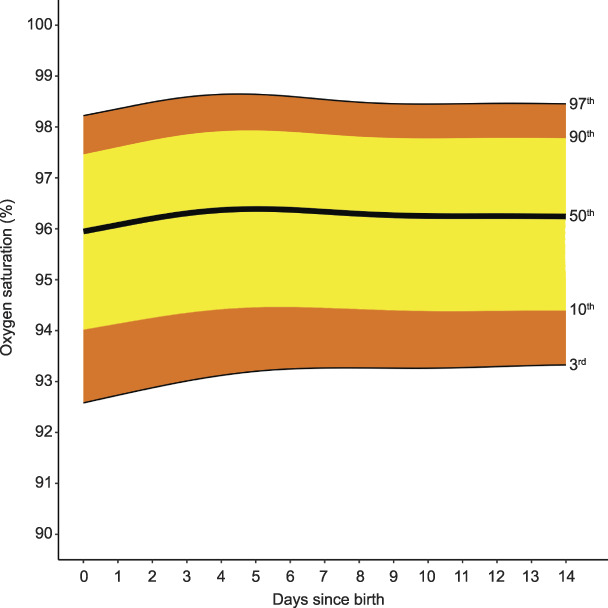
Smoothed centiles for oxygen saturation (%). Day 0 indicates day of birth. Green. Postpartum Vital Sign Reference Ranges. Obstet Gynecol 2021.

**Fig. 6.**
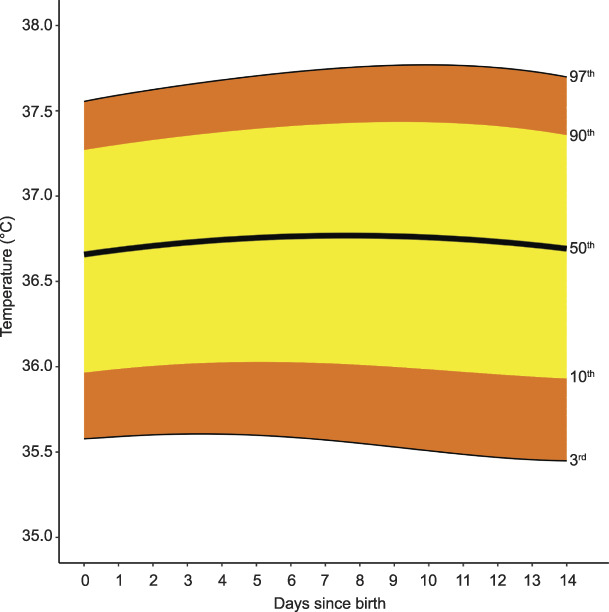
Smoothed centiles for temperature (°C). Day 0 indicates day of birth. Green. Postpartum Vital Sign Reference Ranges. Obstet Gynecol 2021.

Respiratory rate remained unchanged through the postpartum period from the median day-of-birth respiratory rate of 15 breaths per minute (3rd–97th centile 10–22) (Fig. 4). Grouped-day postpartum-specific values for respiratory rate centiles (from tapping method), with the methodology and centiles for respiratory rate derived from the photoplethysmography waveform, are in Appendix 6, available online at http://links.lww.com/AOG/C171. Median, or 50th centile, oxygen saturation was 96% (3rd–97th centile 93–98) on the day of birth, with no clinically significant difference to day 14 (Fig. 5). Median, or 50th centile, temperature did not change from the day-of-birth value of 36.7°C (3rd–97th centile 35.6–37.6) (Fig. 6).

For each vital sign measured, postpartum-specific values for the smoothed centiles and a smoothed day postpartum–specific centile plot with associated 95% CI can be found in Appendix 6 (http://links.lww.com/AOG/C171). Applying the “restrictive” population definitions reduced the cohort to 550 of 909 women (Appendix 7, available online at http://links.lww.com/AOG/C171). There were no clinically significant differences in vital sign reference ranges between the pragmatic and restrictive populations. Although systolic and diastolic blood pressure centiles were numerically slightly lower in the restrictive cohort (higher blood pressures having been excluded) the relatively narrow CIs mainly overlapped.

Median heart rates from 408 nulliparous women were mainly 3–6 bpm higher (with relatively tight CIs that did not overlap) than those from the 501 parous women (Appendix 8, available online at http://links.lww.com/AOG/C171). There were no differences between nulliparous and parous women in other vital signs.

Median heart rates were around 2 bpm higher (with CIs that did not overlap) in the 226 women who received epidural analgesia in the second week after birth compared with those who did not (683 women, Appendix 9, available online at http://links.lww.com/AOG/C171). Similarly, median heart rates were 3–4 bpm higher in the 429 women who received either an epidural or a general anesthetic throughout the 2 postpartum weeks in comparison with those who did not (480 women, Appendix 10 [Appendix 10 is available online at http://links.lww.com/AOG/C171]). There were no differences in other vital signs.

## DISCUSSION

Our multicenter study includes longitudinal data from more than 900 women from whom we produced evidence-based postpartum day–specific centiles for vital signs for the 2 weeks after birth. Blood pressure rose after birth until around days 5–6. The rises in median systolic and diastolic blood pressure are similar to those found in small studies from more than 30 years ago (Walters et al. Lancet 1987;2(8554):330. doi: 10.1016/s0140-6736(87)90912-3).^15,38^ These are suggested to result from mobilization of extracellular fluid and sodium accumulated during pregnancy.^39^ However, these studies did not describe the reference ranges associated with the rise. After the day of birth, the 3rd centile for systolic blood pressure was never less than 97 mm Hg in the full or restrictive populations. This is substantially higher than the 90 mm Hg systolic blood pressure threshold used to recognize sepsis in and after pregnancy^40,41^ and the trigger for escalation in modified obstetric early warning score charts.^16–18,42^ Whether increasing the threshold could improve earlier detection of deteriorating women requires investigation. In our full population, more than 3% of women had systolic blood pressures higher than 140 mm Hg between days 3 and 8. In both the full and restrictive populations, the 97th centile for diastolic blood pressure was higher than 90 mm Hg on days 2–9 postbirth.

These values are higher than the recommended diagnostic or treatment threshold of higher than 140/90 mm Hg as recommended by the International Society for the Study of Hypertension in Pregnancy.^43^ Our normative values are for a single measurement of blood pressure, as we showed previously there are no clinically meaningful population differences between first and second measures of vital signs in pregnancy.^25^ Though high readings would be repeated before making a diagnosis of hypertension, our findings support the higher postpartum treatment thresholds of 150/100 mm Hg or higher recommended by the U.K. National Institute for Health and Care Excellence.^44^

In our previous systematic review, we could not find reliable estimations of the outer centiles of heart rate postpartum.^6^ In our previous work with the 4P cohort, heart rates higher than 100 bpm occurred in more than 10% of women at 40 weeks of gestation.^25^ Postpartum heart rates of greater than 100 bpm were much less common, with the 90th centile for heart rate 100 bpm or less from day 1 postpartum. The 97th centile decreased from 110 bpm on the day of birth to 102 bpm by day 7. These findings suggest that thresholds of greater than 100–120 bpm as a moderate-risk threshold for modified obstetric early warning score escalation^16,17,42^ may be too wide postpartum.

Postpartum thresholds for respiratory rates are poorly described. The median day-of-birth respiratory rate of 15 (3rd–97th centile 10–22) breaths per minute is unchanged from the rate seen in the antenatal 4P cohort.^25^ As during pregnancy, our work shows that a respiratory rate of greater than 22 breaths per minute (as used in the qSOFA [quick Sepsis Related Organ Failure Assessment] tool^45^) would occur in fewer than 3% of observations postnatally, suggesting that this threshold could reasonably translate from other medical practice. Current moderate-risk and high-risk thresholds for respiratory rate of 21–24 and 25 breaths per minute or more (as advocated by the U.K. Sepsis Trust,^41^ IMEWS^17^ and the Scottish Patient Safety Programme^42^) may more accurately identify women at risk of sepsis than moderate-risk^16^ and high-risk^16,18^ thresholds of 21–30 and more than 30 breaths per minute.

The median postpartum oxygen saturation is persistently close to 96%, consistent with the end of pregnancy in the 4P cohort.^25^ As during pregnancy, oxygen saturation less than 93% is an abnormal finding after birth. Because 94% is around the 10th centile throughout the 14 days postpartum, alerting thresholds currently in use of less than 95%^16–18^ or 94% or less^42^ will commonly cause alerts in healthy women.

There was no clinically significant temperature variation postpartum, with median temperatures very similar to those found during pregnancy.^25^ However, the 97th centile was up to 37.8°C postpartum rather than 37.5°C in pregnancy, still lower than the high-risk threshold for escalation of 38°C or higher^16,17,42^ used in many modified obstetric early warning scores. As in pregnancy, the lower 10th centile for temperature lies between 35.9°C and 36.0°C. The lower limit moderate-risk escalation thresholds of less than 36°C suggested for sepsis^41^ would therefore cause an alert to be raised in around 10% of healthy women.

Although higher blood pressures were excluded from the restrictive cohort, blood pressure differences from the pragmatic cohort were marginal. In the nulliparous, epidural, and anesthesia subgroups, heart rates were marginally higher than in the respective comparator cohorts. All other vital sign centiles were similar between comparator subgroups. From these data, it seems unlikely that different alert thresholds for vital signs would be required for any particular subgroup in clinical practice.

The 4P study had secondary objectives of developing a centile-based early warning score and investigating new patterns in vital sign data that will be explored in further work. Our study population is of similar age to the U.K. national average for pregnancy^46^ and with similar BMIs as previous U.K. findings.^47^ Women were predominantly of White European ethnicity (85.2%), equivalent to the most recent England and Wales census data (86%)^48^). Our population therefore appears representative and applicable to clinical practice. Extending to an international population would improve external validity for other settings. By using standardized pregnancy-specific equipment to collect data prospectively from three sites, we are confident our reference ranges are robust.

We present centiles as recommended by the World Health Organization, from a healthy but not overly selected pregnant cohort to provide reliable data for clinicians in clinical practice. These reference ranges can facilitate earlier recognition of unwell postpartum women to reduce morbidity and ultimately minimize maternal mortality. In conjunction with our published antenatal^25^ and planned intrapartum reference ranges, our study will help inform construction of a time-specific modified obstetric early warning score system for use throughout pregnancy and the puerperium.

**Authors' Data Sharing Statement**

Will individual participant data be available (including data dictionaries)? *Yes.*
What data in particular will be shared? *Individual participant data that underlie the results reported in this article, after deidentification (text, tables, figures, and appendices).*
What other documents will be available? *Study protocol, case report forms, standard operating procedures, consent form.*
When will data be available (start and end dates)? *Data will be available between 3 and 36 months after publication.*
By what access criteria will data be shared (including with whom, for what types of analyses, and by what mechanism)? *Researchers who present a sound analysis plan for any valid research will apply by contacting the corresponding author. The validity of proposals will be assessed by the Kadoorie Critical Care Research Group Data Access Committee (which comprises independent researchers, clinicians, patient, and public representatives). Data will be provided using the Group's current compliant system.*
